# Effect of occlusal splint on oxidative stress markers and psychological aspects of chronic temporomandibular pain: a randomized controlled trial

**DOI:** 10.1038/s41598-020-67383-x

**Published:** 2020-07-03

**Authors:** Iva Z. Alajbeg, Ema Vrbanović, Ivana Lapić, Ivan Alajbeg, Lea Vuletić

**Affiliations:** 1 Department of Removable Prosthodontics, School of Dental Medicine, University of Zagreb, Gundulićeva 5, 10 000 Zagreb, Croatia; 2 Medical Biochemistry and Laboratory Medicine, Department of Laboratory Diagnostics, University Hospital Centre Zagreb (KBCZ), Zagreb, Croatia; 3 Department of Oral Medicine, School of Dental Medicine, University of Zagreb, Zagreb, Croatia; 4 Department of Physiology, School of Dental Medicine, University of Zagreb, Zagreb, Croatia; 5 Department of Dentistry, University Clinical Center Zagreb, Zagreb, Croatia

**Keywords:** Biomarkers, Diseases, Health care, Medical research, Signs and symptoms

## Abstract

Temporomandibular disorders (TMD), when progress to a chronic state, might contribute to psychosocial or psychological distress. This study aimed to evaluate the effect of stabilization splint (SS) therapy on pain, pain-related disability and psychological traits of chronic TMD patients, as well as to assess selected oxidative stress (OS) biomarkers during 6-month treatment and associate them with the symptoms of anxiety and depression. Thirty-four participants were randomly assigned into two treatment groups [SS and placebo splint (PS)]. Primary outcomes were pain intensity and pain-related disability while secondary outcomes included depressive and anxiety symptoms. The influence of the treatment type was analyzed with regards to the levels of OS biomarkers in saliva. Participants treated with SS demonstrated significantly greater improvement in pain-related disability (Pain-free mouth opening: p = 0.018, η^2^ = 0.166; Number of disability days: p = 0.023, η^2^ = 0.155) and greater reduction of depressive symptoms scores (p = 0.007, η^2^ = 0.207). When compared to the PS group, participants in the SS group showed a significant reduction of oxidant/antioxidant ratio (p = 0.018, η^2^ = 0.167) at a 3-month follow-up. A stabilization splint provides advantages over PS in the treatment of depressive symptoms and pain-related disability. Furthermore, clinical success in terms of reduction of depressive symptoms, which correlates with the reduction of oxidative stress markers in the SS group, indicates that oxidative stress is related to psychological factors in chronic TMD patients.

## Introduction

Temporomandibular disorders (TMD) encompass the group of disorders affecting jaw joints and masticatory muscles. With the exclusion of pain originating from teeth, TMD is the most common cause of orofacial pain. Their etiology is not clear; however, is considered to be multifactorial: genetic/biologic, behavioral, environmental, socioemotional, and cognitive factors seem to play a role^1,2^. Chronic craniofacial pain, similar to other chronic pain conditions, may be approached from the perspective of the biopsychosocial model that views the pain as a dynamic interaction among and within the biological, psychological, behavioral and social factors unique to each individual^3^. The goal of such an approach to both assessment and management of pain is to tailor the treatment to the specific needs of the individual as standard treatment protocols may lack efficiency if any of these components are ignored^4,5^.

Studies implicated higher occurrence of affective and psychosocial distress, somatic awareness, and pain catastrophizing in patients affected with TMD than in controls^6–10^. Positive correlations were also shown between scores on measures of psychological distress and reported TMD pain and pain-related disability^7,11,12^. Thereby, pain may be a direct cause of emotional distress and lowered quality of life and aid in the development or deterioration of certain psychopathological conditions; on the other hand, individual's pre-existing psychological, behavioral and emotional characteristics may predispose for altered or even enhanced response to painful stimuli^3–5^. Psychological traits, such as depression and anxiety, have been shown to predict new-onset TMD pain^13^ as well as long-term persistence of TMD pain in patients with existing TMD^3,14,15^. Expectedly, initial treatment of patients with TMD may include psychosocial interventions targeted towards changing thoughts, behaviors and/or feelings that may exacerbate pain symptoms which have shown some benefit in treating chronic orofacial pain^16^. Costa et al.^17^ reported that the use of occlusal splints, alongside counseling, may provide an additional effect on the improvement in anxiety and depression symptoms in patients with TMD pain as well as reduce pain catastrophizing. Even though intraoral stabilization appliances for managing TMD are widely used, there is still controversy regarding their efficacy in reducing pain^18^. Therefore, one of the aims of our study was to evaluate the effect of a stabilization splint (SS) in comparison to a placebo splint (PS) on the characteristics of TMD pain and the psychological traits of an individual (anxiety and depression).

It has been suggested that oxidative stress (OS) plays a role in TMD and the experience of pain related to TMD^19,20^. It has also been proposed that increased OS may be associated with the pathogenesis of depression^21^ and anxiety disorders^22^. Although OS has been implicated in the pathophysiology of many diseases due to the observed associations between OS and clinical symptoms^23^, whether OS is the cause or consequence of a disease (or perhaps both) remains elusive. Another aim of our study was to assess selected OS biomarkers during treatment with a stabilization splint in comparison to a placebo appliance and to correlate OS markers with the symptoms of anxiety and depression.

## Methods

### Enrollment

**Figure 1:**
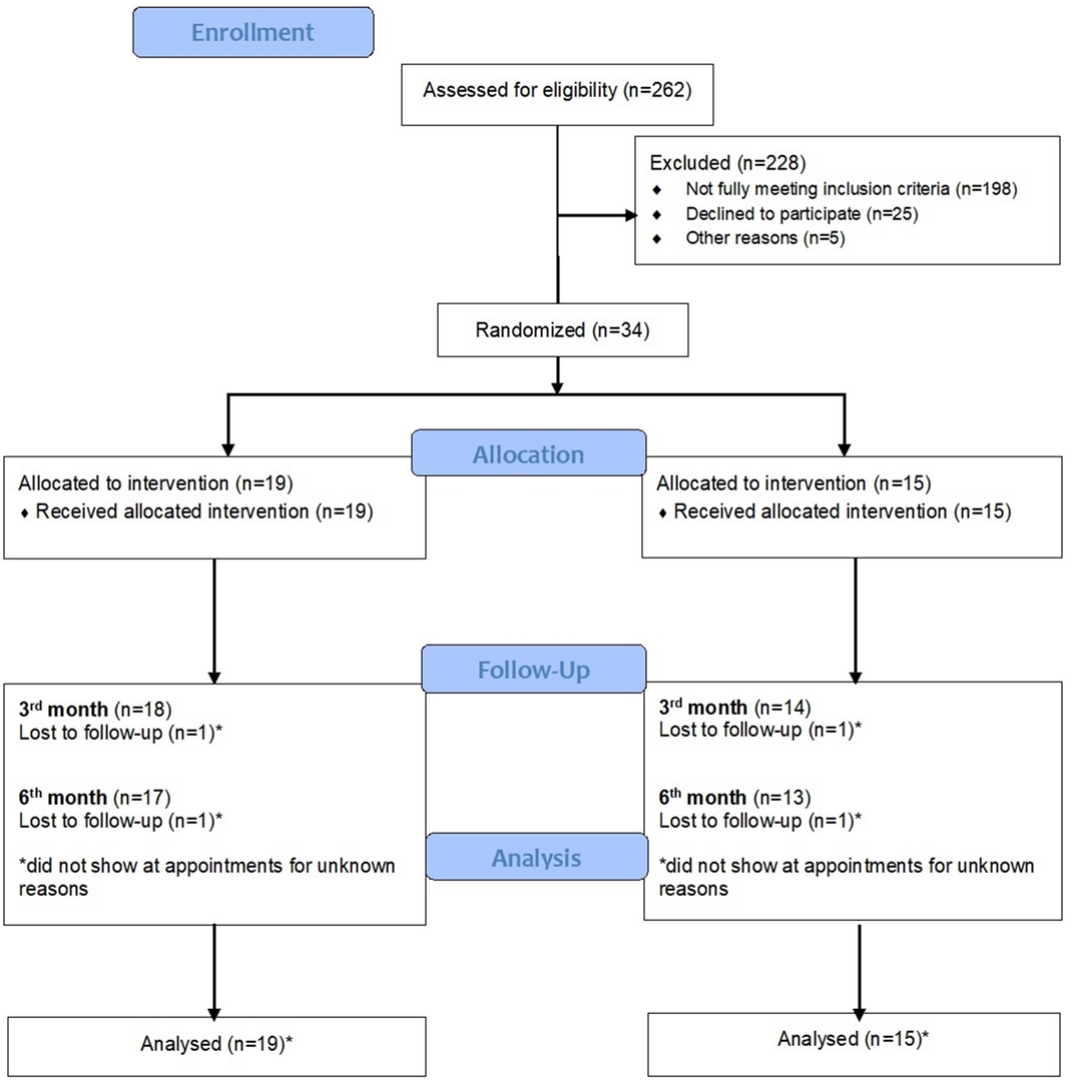
Distribution of the participants throughout the study.

We recruited participants between October 2016 to January 2019, and the final assessment session took place in July 2019. In that period 262 patients seeking treatment for orofacial pain were referred to our research team at the School of Dental Medicine, University of Zagreb. During the recruitment 198 of them did not fully meet the inclusion criteria, 25 of them refused to take part in the study due to travel difficulties and obligations that prevented them from participating, and 5 dropped out due to other reasons. Finally, 34 patients were randomized. The demographic data of participants who were referred but did not fully meet the inclusion criteria were not collected. The flow of participants through each stage of the trial is presented in a CONSORT diagram (Fig. 1). The study was approved by the Ethics Committee of School of dental medicine, University of Zagreb (01-PA-26-6/15; item 3.2) and conducted following the ethical standards of the Helsinki Declaration. All subjects provided their written informed consent before any study procedure was conducted. This study was a part of a 4-year project “The role of oxidative stress and opiorphin in temporomandibular disorders” registered on 24/01/2017 at ClinicalTrials.gov NCT03029494.

Participants were selected among the patients referred to the Department of Removable Prosthodontics and Department of Oral Medicine due to reported pain and discomfort of the temporomandibular region as the primary problem. Participants were eligible if they met criteria for TMD according to Diagnostic Criteria for Temporomandibular Disorders (DC/TMD) with either myofascial pain or arthralgia, had average pain in the last 10 days > 30 mm on a Visual Analogue Scale (VAS), with pain duration of at least 6 months. They had to have their natural teeth and maintain good oral hygiene. Participants were not suitable if they were experiencing (1) inflammatory joint disease, (2) orofacial pain unrelated to TMD, (3) chronic systemic diseases (i.e. diabetes, cardiovascular diseases, malignancies, and autoimmune diseases), (4) causes of headache, unrelated to TMD, listed in the International Classification of Headache Disorders (ICDH II), (5) periodontitis, oral lesions and gum swelling. Individuals who had already been under treatment for TMD were excluded. Finally, participants who use supplements and medication known to affect the oxidative status, and smoke were not able to take part in the study.

### Study design

#### Allocation

To reduce the waiting time and potential withdrawal until a sufficient number of participants are involved, we have chosen a randomization procedure based on our experience of enrolling 6–8 participants in 2 months. The patients were assigned into two treatment groups utilizing random block randomization with a block size of 8 and 6. Randomization was computer-generated, and participants were randomly assigned to two treatment arms, SS or PS. The allocation was prepared by the study's statistician prior to enrollment of the participants.

#### Blinding

Participants were unaware of the differences of each intervention. The principal investigator (IZA), who conducted the baseline evaluations, was the only person aware of the participants’ treatment conditions. All follow-up assessments were performed by the investigator (EV), blinded to the group allocation.

#### Observer training

Ten randomly selected subjects, different from the ones included in the investigation, underwent repeated clinical examinations by two experienced examiners to assess signs and symptoms of TMD (according to DC/TMD). No significant differences were recognized between the first and the second measurements (p = 0.87–0.89, paired t-test). The weighted kappa statistics showed adequate agreement between the observers (κ = 0.86–0.88).

### Interventions

#### Stabilization splint

Full-coverage hard acrylic upper stabilization appliance (Resilit-S, Erkodent, Siemensstraße 3, 72285 Pfalzgrafenweiler, Germany) was made on stone cast mounted in ARTEX articulator in the referent position of centric relation, with a thickness of 1.5 mm at the level of the first molar. Coverage of the approximately 1/3 of the labial and buccal surfaces of the maxillary teeth provided retention for the splints.

All appliances were fabricated in a dental laboratory by the same dental technician. The occlusal surface was smooth and flat, with a canine guidance occlusal scheme. The appliances were adjusted by the clinician (IZA) so that the antagonistic teeth occluded simultaneously with the splint surface, and if necessary, further adjusted at follow-up appointments.

#### Placebo splint

The success of stabilization splint is attributed to various factors such as changes in condylar position, changes in neuromuscular activity, and increase in the vertical dimension of occlusion which probably contributes to the relaxation of the musculature and the relief of temporomandibular joints^24,25^. Therefore, a thin transparent foil with a thickness of 0.5 mm (Erkodent) was used as a placebo splint. The increase in vertical dimension was less than 0.5 mm, therefore thought to provide a negligible influence on occlusion and condylar position. Contacts that interfered with maximal intercuspation were removed.

Patients were instructed to wear their splints only at night while sleeping. During the entire study, participants did not receive other forms of treatment.

#### Adverse events

Patients were monitored for new symptoms or exacerbation of symptoms related to TMD throughout the study. None of the participants experienced any treatment-related adverse effects or pain unrelated to TMD. If this had occurred, we would have considered excluding the participant from the study.

### Procedure

After the patients had been provided with a stabilization splint or a placebo splint, they were followed for 6 months. Primary and secondary outcome data, as well as saliva samples, were collected at baseline, 3-month follow up and post-treatment (6-month follow up). Saliva samples were collected at the same time of the day, at 7 AM and 5 PM. The morning and afternoon sampling was conducted because some of the studies urge to take into consideration the circadian rhythm of markers^26^. We expected changes in oxidative status during the day due to environmental effects and extrinsic stressful events. Also, when validating the method in our previous study, some of the markers (i.e. afternoon MDA) showed better repeatability at a certain time of a day^27^.

Participants were strongly advised to follow detailed sampling instructions provided by the researcher (EV) in person and received those same instructions in writing. The morning samples were collected at home before tooth brushing. Participants were instructed not to eat or drink before morning saliva sampling and not to consume food or drink anything but water 2 h before afternoon sampling. Samples were collected after rinsing the mouth with water. Participants were instructed not to brush or floss their teeth before sampling to avoid blood contamination. Five mL whole, unstimulated saliva was collected into a calibrated tube while the patient was in a normal sitting position. Saliva aliquots (1 mL) were stored at − 80 °C before analysis.

The sample collection and storage principles used in the study are described in detail by Alajbeg et al. ^27^.

### Measures

#### Primary outcome measures

##### Average pain intensity

To assess the average pain, participants marked their average facial pain intensity in the last 30 days on an 11-point scale, where 0 indicated no pain and 10 indicated pain as bad as could be^28,29^.

##### Worst pain intensity

To assess the worst pain, participants filled an 11-point scale indicating the worst intensity of facial pain experienced in the last 30 days (0 = no pain; 10 = pain as bad as could be)^28,29^.

##### Number of disability days

The number of disability days is defined as the number of days of pain that is serious enough to keep the participant from doing normal (usual) activities such as work, school or housework within 1 month.

All three items are part of the Graded Chronic Pain Scale. The instrument was validated based on a 6-month and 1-month time frame and has been extensively used across multiple disorders, languages, and settings^28,29^.

##### Maximum comfortable mouth opening

A maximum comfortable mouth opening (pain-free mouth opening) is defined as the maximum distance the participant could open their mouth without feeling additional pain or discomfort. It was measured as the maximum distance between the incisal edges of maxillary and mandibular central incisors^30^.

#### Secondary outcome measures

##### Anxiety

Generalized Anxiety Disorder (GAD-7) was used for measuring the severity of anxiety symptoms of the participants. The questionnaire consists of 7 statements assessing anxious mood and behavior. Participants selected one of four response choices ranging from 0 (not at all) to 3 (nearly every day), with a total score being the sum of responses on the 7 items on how often they have been bothered by the specified problems. Scores range from 0 to 21, with cut-points 5, 10, and 15 represent mild, moderate, and severe anxiety respectively. The internal consistency of the GAD-7 is excellent (Cronbach α = 0.92)^31,32^. Cronbach’s α in our sample was 0.81 indicating good reliability.

##### Depression

The Patient Health Questionnaire-9 (PHQ-9) was used for measuring the severity of depressive symptoms. The questionnaire comprises 9 items assessing depressed mood, and participants had to indicate on a scale from 0 (not at all) to 3 (nearly every day) how often they have been bothered by the specified problems. Scores range from 0 to 27, with cut-points 5, 10, 15, and 20 represent mild, moderate, moderately severe and severe depression, respectively. The questionnaire has excellent internal reliability with a Cronbach's α of 0.89^33^. In the present study, Cronbach's α was 0.75, indicating acceptable internal consistency.

#### Biochemical stress markers

Uric acid (UA) was determined using an automated enzymatic two-step colorimetric assay by Roche Diagnostics, Mannheim, Germany. Firstly, uric acid is cleaved by uricase to allantoin and hydrogen peroxide. In the presence of peroxidase, hydrogen peroxide oxidizes the chromogen 4-aminophenazone to form a quinone-diimine dye which increases absorbance that is measured at 293 nm. Within-run and between-run coefficients of variation are 1.4% and 1.8%, respectively, as determined during method verification following the Clinical and Laboratory Standards Institute EP15-A3 protocol (Clinical and Laboratory Standards Institute). UA was measured on the Cobas c501 automated biochemistry analyzer (Roche Diagnostics, Mannheim, Germany).

Superoxide dismutase (SOD) and total antioxidant capacity (TAC) were measured using commercially available reagent kits RANSOD and TAS (Randox Laboratories Ltd., Crumlin, United Kingdom) which were used according to instrument-tailored applications defined by the manufacturer, on Cobas c501 biochemistry analyzer (Roche Diagnostics, Mannheim, Germany).

The RANSOD assay utilizes xanthine and xanthine oxidase that generates superoxide radicals which react with 2-(4-iodophenyl)-3-(4-nitrophenol)-5-phenyltetrazolium chloride to form a red formazan dye. The activity of SOD is measured by the degree of inhibition of this reaction, where one unit of SOD corresponds to a 50% inhibition of the reaction rate which is measured at 505 nm. According to the manufacturer, within-run and between-run CVs are 4.6% and 7.1%, respectively.

TAS assay is based on simultaneous incubation of a radical generating system consisting of metmyoglobin and hydrogen peroxide, which oxidizes the ABTS substrate and subsequently produces ABTS cation radicals, which are measured photometrically at 600 nm. The presence of antioxidants in the sample cause suppression of this reaction to a degree that is proportional to their concentration. Within-run CV was determined by 10 replicate measurements of a saliva sample and was 2.8% at 1.5 mmol/L TAC.

Malondialdehyde (MDA) was determined using the MDA Adduct competitive ELISA kit (Kamiya Biomedical Company, Seattle, USA) that quantifies MDA-stable hybrid protein adducts (MDA-adducts), formed during lipid peroxidation. The ELISA microplate is precoated with an MDA conjugate. The anti-MDA polyclonal antibody competes for binding between MDA adducts in the sample and MDA conjugate bound on the wells of the ELISA microplate. The addition of HRP-conjugated secondary antibody produces a color reaction that is measured spectrophotometrically at 450 nm. The content of MDA adducts in the sample is inversely proportional to the intensity of the colorimetric reaction and is determined from a calibration curve. The content of MDA adduct in the calibrators is predetermined by a Thiobarbituric Acid Reactive Substances (TBARS) kit. A within-run CV of 16.6% at the concentration of 134 pmol/mL was obtained by 10 repeated analyses of one saliva sample in a single batch.

All measured OS markers were normalized to the concentration of total protein.

Total proteins (TP) in saliva were quantified using the commercially available turbidimetric assay TPUC3 (Roche Diagnostics, Mannheim, Germany) for measurement of total proteins in urine and cerebrospinal fluid that exhibits satisfactory measuring range for saliva samples, i.e. from 0.02 to 2 g/L. Proteins in the sample react with benzethonium chloride in a basic medium producing turbidity that is measured photometrically at 505 nm. Within-run and between-run CVs are 0.9% and 1.0%, respectively, and were also obtained during method verification. TP were measured on the Cobas c501 automated biochemistry analyzer (Roche Diagnostics, Mannheim, Germany).

The salivary oxidative index ratio was used to reflect the changes in oxidative balance. It was calculated by dividing the concentration of pro-oxidant MDA by the concentration of the antioxidant SOD (MDA/SOD ratio).

### Analytic plan

#### Sample size determination

Since there are no similar published studies on the effect of stabilization splint compared to placebo splint on the level of oxidative stress markers, the sample size calculation was based on the studies that compared the effect of a stabilization splint on biochemical markers with no-treatment group^34^. The mean changes in the salivary MDA level at a 3-month follow-up were estimated to be 0.13 µM and 0.08 µM, respectively (SD 0.05 µM). A sample size calculation for a repeated measurement within-between analysis of variance with two groups showed that 26 participants (13 per group) were required to obtain a power of 0.80 at an alpha level of 0.05.

Modeled from previous studies which compared stabilization splint with other types of treatment (only counseling)^17^, a-priori power analyses based upon 5-month reduction in pain catastrophizing (1.01, 1.3, respectively, estimated SD 0.28) revealed that 24 participants (12 per group) were necessary to achieve 80% power with a significance level of 0.05.

If these calculations can be generalized and applied to our sample and design, the size of our sample (n = 34) provided acceptable power to identify moderate treatment effect size differences.

#### Analyses

Data on all patients who were randomly assigned were analyzed on an intention to treat (ITT) basis. Once enrolled, retention at follow-up assessments or the post-treatment was 89% (n = 30, SS = 17, PS = 13). We substituted missing data employing the K-Nearest Neighbour imputation algorithm.

All analyses were performed using Statistica 13.4.0 software package (1984–2018 TIBCO Software Inc.). The distribution of data was tested using the Shapiro–Wilk test. Before performing the statistical analyses, a log transformation was performed for all data that were not normally distributed (OS markers). We first performed a series of Student's t-tests and chi-square tests to determine whether there were any differences at baseline on demographic and pain variables as well as on oral behaviors between the two randomized groups (SS, placebo).

Additional analyses were carried out to evaluate whether: (1) treatment groups differed significantly at baseline, (2) outcome variables were correlated significantly at baseline.

Analyses of differences between groups at 3-month follow-up and post-treatment were tested using ANCOVA to determine whether the follow-up and post-test means, adjusted for pre-test scores, differ between the two groups. Baseline variables (i.e., average pain, the worst pain, GAD-7, PHQ-9, pain-free mouth opening and levels of OS markers) were included as covariates.

Within-group changes for SS and PS on primary and secondary treatment outcomes at both 3-month follow-up and post-treatment were analyzed utilizing Within subjects repeated measures ANOVA. Bonferroni corrected post hoc tests were used to evaluate the difference between the time points. Partial eta squared (η^2^) was calculated for analyses as an indication of the effect sizes.

If the sphericity assumption was violated (Mauchly’s test of sphericity p < 0.05), Greenhouse–Geisser corrections were performed.

Associations between changes in treatment outcomes were tested using Pearson's correlation.

## Results

### Participants characteristics

A total of 64 participants met initial criteria, and 34 agreed to participate in the study (mean age 36.1 ± 11.95). They were randomized to one of the two treatment groups (SS = 19, PS = 15). All participants were female and Caucasian (n = 34) (see Table 1 for demographic information). Time since pain onset varied between 6 and 24 months. More than 65% of participants reported TMD-related pain in more than one area and 47% of participants reported headaches related to TMD. Eleven participants (32.4%) reported experiencing pain every day in the last six months, 10 (29.4%) reported pain on most days, and 13 (38.2%) reported pain in about 1–3 days per week. Randomization resulted in a balanced distribution in treatment groups at baseline, according to all conditions, with no significant group differences in terms of demographics and pain variables. Oral behaviors evaluation, measured with the Oral Behaviors Checklist, revealed that high frequency of oral parafunctions was reported in 55.9% of the subjects, with no significant differences between groups. Also, treatment groups did not differ at baseline on either primary or secondary outcomes (anxiety or depressive symptoms) (Table 2).

**Table 1:** Participant baseline characteristics.

**Measure**	**Stabilization splint (n = 19)**	**Placebo splint (n = 15)**	**Total (n = 34)**
Demographics			
Age, mean (SD)	38.8 (11.84)	32.6 (11.54)	36.1 (11.95)
Ethnicity			
Caucasian, n (%)	19 (100%)	15 (100%)	34 (100%)
Marital status			
Never married, n (%)	4 (21.05%)	5 (33,33%)	9 (26.47%)
Living as married, n (%)	5 (26.31%)	1 (6,67%)	6 (17.64%)
Married, n (%)	7 (36.84%)	8 (53,33%)	15 (44.12%)
Divorced, n (%)	2 (10.53%)	1 (6,67%)	3 (8.82%)
Widowed, n (%)	1 (5.26%)	–	1 (2.94%)
Level of education			
High school, n (%)	5 (26.32%)	7 (46.67%)	12 (35.29%)
Some university, n (%)	4 (21.05%)	4 (26.67%)	8 (23.53%)
University degree, n (%)	7 (36.84%)	3 (20.00%)	10 (29.41%)
PhD, n (%)	3 (15.79%)	1 (6.67%)	4 (11.76%)
Pain variables			
Time since pain onset [months], mean (SD)	12.9 (7.5)	13.4 (6.9)	13.2 (7.1)
Pain in more than one area, n (%)	13 (68.42%)	10 (66.67%)	23 (67.65%)
Headache related to TMD, n (%)	9 (47.37%)	7 (46.67%)	16 (47.06%)
Pain duration [days/6 months], mean (SD)	112 (62)	94 (67)	104 (63)
Oral behaviors			
Normal behaviors, n (%)	2 (10.6%)	2 (13.3%)	4 (11.8%)
Low risk, n (%)	5 (26.3%)	6 (40%)	11 (32.4%)
High risk, n (%)	12 (63.2%)	7 (46.7%)	19 (55.9%)

**Table 2:** Means and SDs of outcome measures for each group at each time point (intention to treat analyses).

	**Stabilization splint**			**Placebo splint**			**Partial eta-squared**^ϕ^	
	**Baseline**	**3-month follow-up**	**Post treatment**	**Baseline**	**3-month follow-up**	**Post treatment**	**Baseline vs** **3-month follow-up**	**Baseline** **vs post-treatment**
**Primary outcomes**								
Average pain intensity	4.68 (2.31)	2.16 (1.80)	1.84 (1.77)	4.53 (1.41)	2. 73(1.58)	2.86 (1.99)	0.032	0.075
Worst pain intensity	6.84 (2.14)	3.47 (2.14)	2.94 (2.37)	5.80 (2.18)	3.93 (1.94)	4.53 (2.80)	0.038	0.106
No of disability days	5 (8)	0.7	0.15	4.2 (8)	3.2	2.1	0.064	**0.155**
Pain free opening (mm)	27.58 (9.88)	34. 84 (9.47)	35.00 (7.72)	27.93 (6.18)	30. 13 (7.31)	28.80 (8.77)	0.101	**0.166**
Secondary outcomes Psychological factors								
Generalized anxiety (GAD-7)	6.74 (5.49)	4. 58 (4.47)	4.10 (4.80)	6.80 (4.54)	5.67 (4.10)	6.40 (3.50)	0.035	0.095
Depression (PHQ-9)	6.68 (5.33)	3.42 (2.75)	3.16 (2.75)	7.00 (5.02)	6.00 (3.05)	6.07 (3.32)	**0.193**	**0.207**
Biochemical stress markers								
log SOD (*morning)* log SOD (*afternoon)*	3.44 (0.33) 3.29 (0.29)	3.21 (0.81) 3.19 (0.81)	3.19 (0.81) 3.13 (0.79)	3.16 (0.93) 3.33 (0.37)	3.37 (0.95) 3.34 (0.28)	3.47 (0.25) 3.76 (0.17)	0.008 0.012	0.059 0.042
log TAC *(morning)* log TAC *(afternoon)*	0.47 (0.25) 0.48 (0.25)	0.32 (0.30) 0.39 (0.24)	0.29 (0.21) 0.36 (0.25)	0.47 (0.25) 0.49 (0.24)	0.38 (0.24) 0.41 (0.19)	0.39 (0.12) 0.41 (0.23)	0.023 0.001	0.099 0.010
log MDA *(morning)* log MDA *(afternoon)*	2.33 (0.72) 2.76 (0.75)^†^	2.24 (0.93) 2.46 (0.89)	2.13 (0.99) 2.62 (0.88)	2.84 (0.80) 3.25 (0.51)^†^	2.70 (0.85) 3.19 (0.49)	2.87 (0.81) 3.30 (0.44)	0.003 0.090	0.053 0.077
log UA *(morning)* log UA *(afternoon)*	2.63 (0.34) 2.66 (0.33)	2.40 (0.73) 2.38 (0.77)	2.38 (0.76) 2.49 (0.69)	2.70 (0.35) 2.65 (0.39)	2.58 (0.37) 2.66 (0.34)	2.67 (0.26) 2.63 (0.40)	0.009 0.061	0.051 0.019
log MDA/SOD *(morning)* log MDA/SOD *(afternoon)*	0.09 (0.15) 0.24 (0.26)	0.09 (0.15) 0.13 (0.14)	0.12 (0.24) 0.21 (0.23)	0.24 (0.27) 0.39 (0.33)	0.12 (0.11) 0.34 (0.25)	0.27 (0.27) 0.35 (0.21)	0.066 **0.167**	0.001 0.042

*GAD-7* generalized anxiety, *PHQ-9* depression, *SOD* superoxide dismutase, *TAC* total antioxidant capacity, *MDA* malondialdehyde, *UA* uric acid.

^†^Statistically significant difference between SS and PS at baseline.

^ϕ^Effect size for treatment differences between SS and PS group.

As expected, several variables were significantly correlated at baseline. Number of disability days was significantly correlated with anxiety (r = 0.339, p = 0.049), worst pain (r = 0.387, p = 0.023) and average pain (r = 0.49, p = 0.002). Anxiety was positively correlated with depressive symptoms (r = 0.715, p < 0.0001). A significant positive correlation was found between log TAC and log UA values (AM: r = 0.514, p = 0.002; PM: r = 0.53; p = 0.001), while morning log MDA and log SOD were negatively correlated (r = − 0.431, p = 0.01). Both anxiety and depression were negatively correlated with afternoon antioxidants levels (r_GAD-7-logTAC_ =  − 0.415, p = 0.014; r_GAD-7-logUA_ = 0.381, p = 0.026; r_PHQ-9-logTAC_ = 0.399, p = 0.019; r_PHQ-9-logUA _ = 0.484, p = 0.004).

### Primary outcome measures changes

Improvement in pain-free mouth opening at a 3-month follow-up was more evident in SS than in the PS group but did not achieve statistical significance at the 0.05 level (F = 3.47, p = 0.07, η^2^ = 0.101). When controlling for baseline values, at post-treatment, participants treated with SS exhibited significantly stronger improvement in pain-free mouth opening than participants who received PS (F = 6.20, p = 0.018, η^2^ = 0.166) (Table 2).

Participants that received SS demonstrated a greater decrease in pain than participants who were treated with PS but the differences between groups were not statistically significant (Table 2). Within-group analyses showed a significant decrease in both average (F = 19.34; p = 0.00003, η2 = 0.52) and worst pain (F = 27.52; p < 0.001, η2 = 0.61) for the SS group, while in PS group a significant decrease in average pain was found (F = 6.55; p = 0.004, η2 = 0.318). The results of post hoc analyses showing differences from baseline to 3rd and 6th month are presented in Table 3.

**Table 3:** Within groups ANOVA—post-hoc pairwise comparisons.

			**Stabilization splint**			**Placebo splint**		
			**Mean differ**	**95% CI**	**p**^**†**^	**Mean differ**	**95% CI**	**p**^**†**^
**Primary outcomes**	**Average pain intensity**	Baseline vs 3-month follow-up	2.526	1.26–3.78	** < 0.001**	1.800	0.38–3.21	**0.009**
		Baseline vs post-treatment	2.842	1.58–4.09	** < 0.001**	1.666	0–25–3.08	**0.016**
	**Worst pain intensity**	Baseline vs 3-month follow-up	3.368	1.93–4.79	** < 0.001**	1.866	− 0.06−3.79	0.06
		Baseline vs post-treatment	3.894	2.46–5.32	** < 0.001**	1.266	− 0.65–3.192	0.31
	**No of disability days**	Baseline vs 3-month follow-up	4.263	0.69–7.83	**0.014**	1.00	− 4.35–6.35	1.00
		Baseline vs post-treatment	4.842	1.27–8.41	**0.005**	2.133	− 3.22–7.48	0.95
	**Pain free opening (mm)**	Baseline vs 3-month follow-up	− 7.263	− 12.18−2.34	**0.002**	− 2.2	− 6.09–1.69	0.48
		Baseline vs post-treatment	− 7.421	− 12.34–2.49	**0.001**	− 0.866	− 4.75–3.02	1.00
**Secondary outcomes** *Psychological factors*	**Generalized anxiety (GAD-7)**	Baseline vs 3-month follow-up	2.157	0.13–4.18	**0.033**	1.133	− 1.08–3.35	0.61
		Baseline vs post-treatment	2.631	0.61–4.65	**0.007**	0.40	− 1.82–2.62	1.00
	**Depression (PHQ-9)**	Baseline vs 3-month follow-up	3.263	1.21–5.31	** < 0.001**	1	− 2.02–4.02	1.00
		Baseline vs post-treatment	3.526	1.47–5.58	** < 0.001**	0.933	− 2.09–3.95	1.00
**Biochemical stress markers**	**log SOD (morning)**	Baseline vs 3-month follow-up	0.238	− 0.17–0.65	0.47	− 0.208	− 0.92–0.50	1.00
		Baseline vs post-treatment	0.262	− 0.15–0.67	0.36	− 0.304	− 1.01–0.41	0.85
	**log SOD (afternoon)**	Baseline vs 3-month follow-up	0.096	− 0.31–0.51	1.00	− 0.010	− 0.23–0.21	1.00
		Baseline vs post-treatment	0.162	− 0.25–0.57	0.97	− 0.044	− 0.27–0.18	1.00
	**log TAC (morning)**	Baseline vs 3-month follow-up	0.151	0.02–0.29	**0.023**	0.086	0.05–0.22	0.35
		Baseline vs post-treatment	0.179	0.05–0.31	**0.005**	0.083	− 0.05–0.22	0.39
	**log TAC (afternoon)**	Baseline vs 3-month follow-up	0.091	− 0.03–0.21	0.19	0.084	− 0.05–0.21	0.33
		Baseline vs post-treatment	0.122	0.01–0.24	**0.046**	0.088	− 0.04–0.22	0.28
	**log MDA (morning)**	Baseline vs 3-month follow-up	0.087	− 0.33–0.51	1.00	0.145	− 0.26–0.55	1.00
		Baseline vs post-treatment	0.201	− 0.22–0.62	0.71	− 0.026	− 0.44–0.38	1.00
	**log MDA (afternoon)**	Baseline vs 3-month follow-up	0.289404999	− 0.10–0.68	0.21	0.066	− 0.21–0.34	1.00
		Baseline vs post-treatment	0.133	− 0.26–0.52	1.00	− 0.041	− 0.32–0.24	1.00
	**log UA (morning)**	Baseline vs 3-month follow-up	0.22	− 0.04–0.49	0.11	0.128	− 0.04–0.29	0.19
		Baseline vs post-treatment	0.25	− 0.02–0.52	**0.049**	0.034	− 0.13–0.20	1.00
	**log UA (afternoon)**	Baseline vs 3-month follow-up	0.286	− 0.09–0.66	0.19	− 0.018	− 0.23–0.19	1.00
		Baseline vs post-treatment	0.171	− 0.21–0.54	0.78	0.017	− 0.19–0.23	1.00
	**log MDA/SOD (morning)**	Baseline vs 3-month follow-up	0.002	− 0.06–0.06	1.00	0.123	0.01–0.23	**0.02**
		Baseline vs post-treatment	− 0.022	− 0.09–0.04	1.00	− 0.024	− 0.13–0.087	1.00
	**log MDA/SOD (afternoon)**	Baseline vs 3-month follow-up	0.106	− 0.01–0.21	0.066	0.058	− 0.12–0.24	1.00
		Baseline vs post-treatment	0.025	− 0.087–0.13	1.00	0.049	− 0.13–0.23	1.00

*GAD-7* generalized anxiety, *PHQ-9* depression, *SOD* superoxide dismutase, *TAC* total antioxidant capacity, *MDA* malondialdehyde, *UA* uric acid, *CI* Confidence Interval.

^**†**^Bolded p-values represent statistically significant differences.

Participants in the SS group demonstrated a greater decrease in the number of disability days both at 3-month follow-up and post-treatment, but the differences between groups reached statistical significance only at post-treatment follow up (F = 5.7, p = 0.023, η^2^ = 0.155) (Table 2).

**Figure 2:**
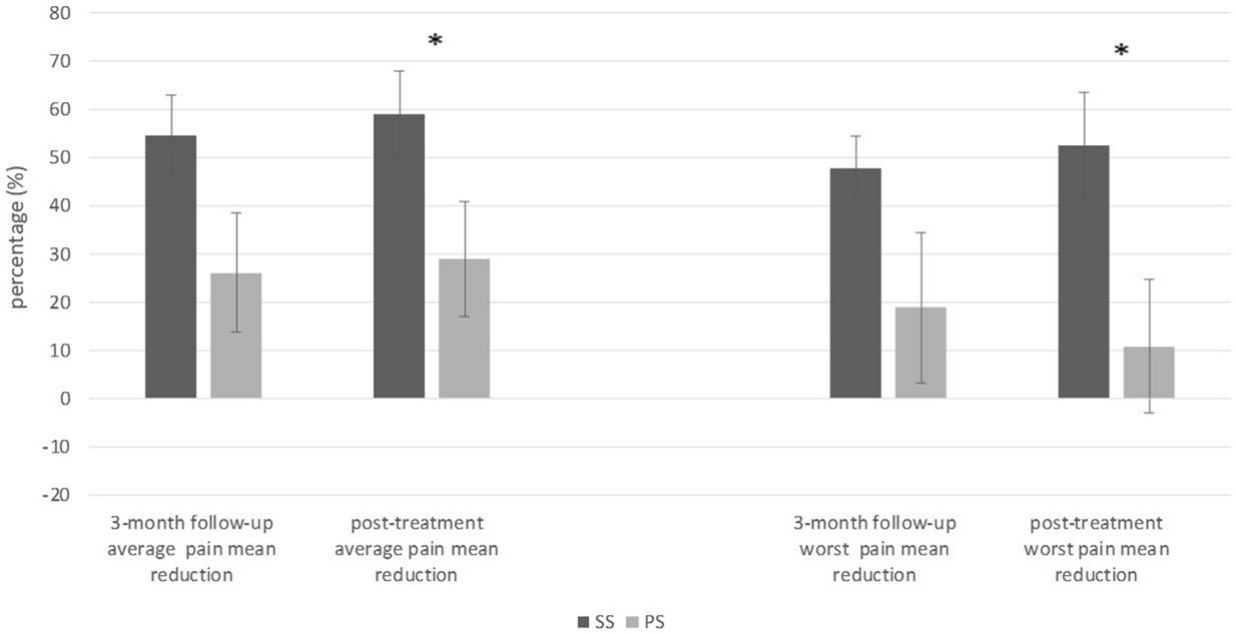
Relative changes of primary outcomes in two treatment groups. The whiskers represent standard errors; the asterisk indicates a statistical significant difference (p < 0.05); *SS* stabilization splint, *PS* placebo splint.

When comparing changes in primary outcomes between groups, a significantly greater reduction of average (t = 2.04, p = 0.04) and worst pain levels (t = 2.36, p = 0.02) at post-treatment, relative to those recorded at baseline, were noted in SS group compared to placebo (Fig. 2).

### Secondary outcome measures changes

While controlling for baseline values, at 3-month follow-up participants in the SS group demonstrated stronger improvements for depressive symptoms than those treated with PS (F = 7.41, p = 0.011, η^2^ = 0.193). When comparing treatment effects at post-treatment to baseline values stronger improvements for depressive symptoms again were present in subjects treated with SS (F = 8.08, p = 0.007, η^2^ = 0.207) (Table 2).

Although participants treated with SS demonstrated stronger improvement than participants treated with PS for anxiety symptoms, the differences between the groups were not statistically significant (Table 2). In examining within-group changes, a significant improvement for anxiety symptoms (F = 6.06; p = 0.018, η^2^ = 0.25) was found only in the SS group The results of post hoc analyses showing differences from baseline to 3rd and 6th month are presented in Table 3.

**Figure 3:**
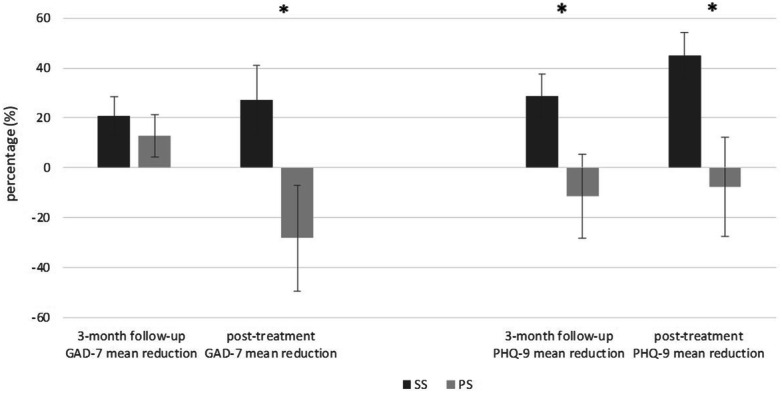
Relative changes of secondary outcomes in two treatment groups. *GAD 7* generalized anxiety, *PHQ-9* depression; the whiskers represent standard errors; the asterisk indicates a statistical significant difference (p < 0.05); *SS* stabilization splint, *PS* placebo splint.

When comparing changes in secondary outcomes between groups, participants in the SS group presented a significantly greater reduction in anxiety (t = 2.25, p = 0.03) and depression (t = 2.54, p = 0.01) at post-treatment relative to values obtained at baseline, compared to placebo (Fig. 3).

### Biochemical stress markers changes

**Figure 4:**
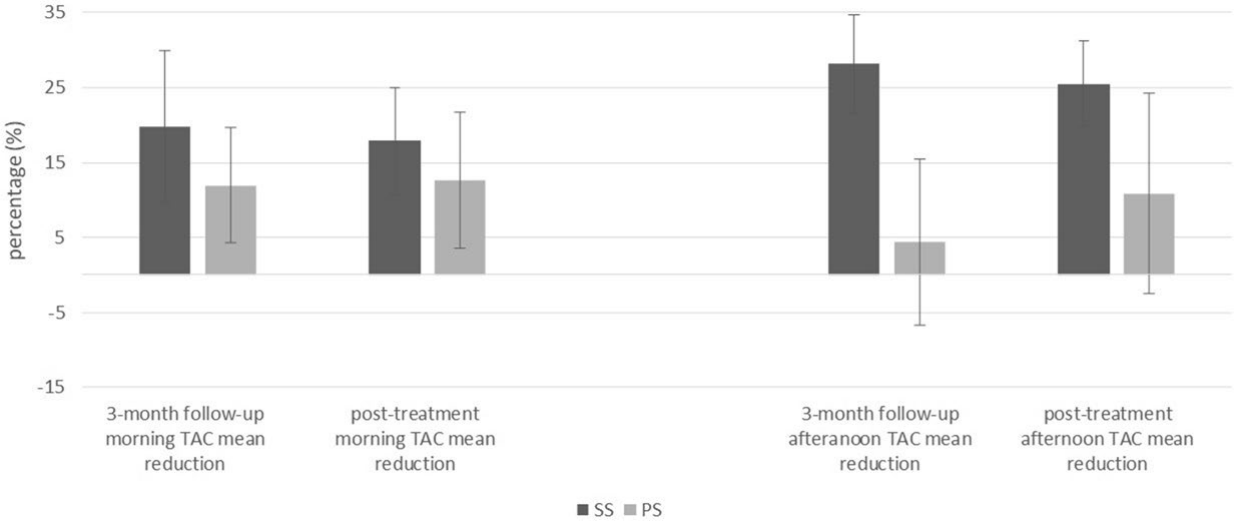
Relative changes of total antioxidant capacity in two treatment groups. TAC: total antioxidant capacity; the whiskers represent standard errors; the asterisk indicates a statistical significant difference (p < 0.05); SS: stabilization splint; PS: placebo splint.

For the biochemical stress markers, although participants who received SS demonstrated a greater decrease in the levels of log TAC, log MDA and log UA than participants who received PS, the differences between the groups were not statistically significant (Table 2). The relative decrease of salivary TAC levels was not significantly different between the groups, although in SS group decrease was greater than those of the PS group (Fig. 4).

However, according to within-group analyses, participants in SS group showed a significant decrease in the levels of log TAC (AM: F = 6.48, p = 0.005, η^2^ = 0.26; PM: F = 3.49, p = 0.042, η^2^ = 0.16) and log UA (AM: F = 3.43, p = 0.043, η^2^ = 0.16) while those in the PS group did not change from baseline to post-treatment follow up (p > 0.05). The results of post hoc analyses showing differences from baseline to 3rd and 6th month are presented in Table 3.

The oxidative stress index ratios in saliva were significantly different between patients treated with SS when compared with those treated with PS. While controlling for baseline values, at 3-month follow-up participants in the SS group demonstrated a greater decrease in the afternoon log MDA/SOD ratio than those treated with PS (F = 6.25, p = 0.018, η^2^ = 0.167) (Table 2).

#### Association between changes in psychological traits and TAC concentration change

At post-treatment the reduction of depressive symptoms was positively correlated with the decrease of afternoon TAC (r = 0.595, p = 0.007) in participants treated with SS. No significant associations were found between a decrease of secondary outcomes and TAC decrease in participants treated with PS (Table 4).

**Table 4:** Correlation between changes in psychological traits and TAC concentrations' change.

**Treatment**	**Reduction**	**3-month follow-up**		**Post-treatment**	
		**Morning TAC**	**Afternoon TAC**	**Morning TAC**	**Afternoon TAC**
**SS**	**GAD-7**	0.152	0.181	− 0.049	0.413
	**PHQ-9**	0.039	− 0.125	0.413	0.596^†^
**PS**	**GAD-7**	− 0.354	− 0.102	− 0.164	0.376
	**PHQ-9**	− 0.392	0.151	− 0.321	0.216

*GAD-7* generalized anxiety, *PHQ-9* depression, *TAC* total antioxidant capacity, *SS* Stabilization splint, *PS* placebo splint.

^†^p < 0.05.

## Discussion

This study aimed to assess if a 6 months long treatment of patients with TMD with a stabilization splint would lead to a greater reduction in pain, pain-related disability (maximal comfortable mouth opening; interference with daily activities) and the expression of depressive and anxiety symptoms in comparison to a placebo splint. The influence of the treatment type was also analyzed with regards to the levels of several OS biomarkers in saliva. Also, levels of salivary OS markers were associated with the psychological characteristics of patients with TMD, which is, to the best of our knowledge, the novelty of this study.

Results of previous studies assessing the effects of hard stabilization appliances on self-reported pain as a measure of TMD severity are inconsistent. However, a meta-analysis demonstrated that there is evidence that hard stabilization splints may offer improvement in TMD pain symptoms in comparison to both control appliances and no treatment^18^. In the present study, between-group comparisons did not confirm the superiority of SS over PS in TMD pain experience. Within-group analyses showed a decrease in average pain for both groups, while decrease in worst pain was present only in the SS group. The absence of statistically significant differences for painful symptoms between groups supports the theory that the placebo has the same therapeutic efficacy as SS. However, given that within-group analysis showed significant changes for the worst pain only in the SS group, we would prefer not to disregard the possibility that the SS could have a superior therapeutic effect over the PS. Studies with a larger sample could clarify this ambiguity regarding our results.

A significant difference between groups was found for pain-free mouth opening and a number of disability days at post-treatment follow up (after 6 months), however with a small effect size. Our results thus indicate that SS may offer slightly greater benefits in reducing pain and pain-related disability. Taking into account that hypotheses proposed to explain biological mechanisms by which oral appliances influence experience of both myofascial pain and arthralgia have not been substantiated by sufficient evidence^35^, PS in our study may have provided some pain relief even though these splints were constructed to have as little effect as possible on changes in both vertical dimension and the position of the lower jaw. It is important to have in mind that the pain-relieving effect of both placebo and stabilization splint may be attributed to other factors rather than merely the change in the vertical dimension and changes in the positioning of the condyle which were the main difference between SS and PS in our study. Those factors (cognition, behavior consciousness, sensorial alterations, muscle rearrangement,) might also lead to pain reduction, thus must be taken into consideration when interpreting our results.

Treatment with SS has led to a greater improvement in depressive symptoms scores than the use of PS, but again with a small effect size. This finding could be suggestive of some beneficial biological effects of SS that might alleviate pain and, consequently, lead to an improvement in patients’ psychological characteristics as no additional form of treatment was used in our study (e.g., patient education and counseling, psychosocial interventions). It must be pointed out that the participants of our study were not patients with diagnosed depression or anxiety disorder. However, it may be expected that individuals suffering from chronic pain express depressive symptoms and more anxious behavior in comparison to pain-free individuals^36^. Thereby, certain signs of psychological distress that relate to these conditions have been found to be more prominent among pain patients (low energy, disturbed sleep, worry) than others (guilt, loneliness)^37^. Average GAD-7 and PHQ-9 scores revealed the presence of, on average, mild anxiety and depressive symptoms in our study sample. These symptoms may be partly related to pain caused by TMD (rated of medium average intensity), but also to other factors such as personality traits and interindividual differences in coping profiles (the way people manage or deal with stressful situations)^38^. Different factors influencing participants' responses to GAD-7 and PHQ-9 might explain why we found a significant difference in depressive symptoms scores between groups, while the difference in anxiety scores was not statistically significant. Taken into account that both SS and PS group expressed similar levels of anxiety and depressive symptoms at baseline (due to randomization) and, thereby, participants were evenly distributed between the groups with regards to their level of adaptation to their long-lasting painful condition, alleviation of pain caused by SS therapy could be expected to influence answers to PHQ-9 and reflect itself in increased pleasure or interest in doing things, better mood, better sleep, better concentration and/or a feeling of having more energy. On the other hand, the same influence of SS therapy would not need to be reflected in the GAD-7 scores in individuals who, as a personality trait, tend to worry too much, who are always in fear that something could go wrong (out of their control) and/or are easily annoyed.

Studies assessing the influence of splint therapy for TMD on OS are scarce. Baş et al.^34^ reported the successful use of stabilization splints in relieving clinical symptoms of TMD but found no effect on the markers of OS (MDA, interleukin 6 and 8-hydroxydeoxyguanosine) in synovial fluid in a 3-month follow up period. A previous study of our research group^39^ demonstrated a significant reduction in afternoon TAC in TMD patients treated with occlusal splints after a 3-month follow up. In addition, a significant decrease in afternoon MDA and MDA/SOD ratio was found in the subgroup of patients with high pain intensity whose pain, expressed through VAS scores, decreased over time. In the present study, statistically insignificant differences between SS and PS group in the levels of all measured OS biomarkers at baseline (except for the afternoon MDA) remained such during the whole treatment period except for the afternoon MDA/SOD ratio that was significantly lower in the SS group at 3-month follow up. Within-group analyses showed a significant decrease in TAC and UA in the SS group. Our previous study on OS indicators in chronic TMD^40^ showed that TAC was significantly higher in TMD patients than in healthy controls. We suggested that higher TAC in patients with TMD pain lasting more than 6 months might imply a compensatory increase of the antioxidant enzymes in response to changing levels of OS as a prerequisite for efficient defense. Based on this explanation, and taken into account that, in the present study, a positive correlation was found between TAC decrease and a decrease in depressive symptoms scores in the SS group, a reduction in TAC levels in patients who used SS could be seen as an indicator of improvement.

The finding that both anxiety and depressive symptoms scores were negatively correlated with afternoon antioxidant levels (TAC and UA) at baseline could be considered as an expected finding. In light of this result, a positive correlation between TAC decrease and a decrease in depressive symptoms scores in the SS group seems difficult to explain. A possible explanation could be in the observed reduction in the MDA/SOD ratio indicating lower oxidative stress and a possible decrease in the activity of other substances that may cause oxidative damage but were not measured in our study. In this way, the reduction of TAC during the course of the therapy might reflect a changing balance between oxidative and antioxidative systems.

In this study, no association between OS markers and pain was found. Our previous study^39^ found that higher TAC levels were related to higher pain intensity so the results of the correlation analyses in the present study could possibly be different if the patients had been stratified according to pain intensity.

The present study has limitations. Regardless of power analysis, we consider that for research on such a delicate and variable matter as OS markers, a larger sample size would provide stronger evidence. The additional benefit in the interpretation of the results could have been achieved by including an additional control group that would receive no treatment. However, it seems unlikely that the participants would readily agree not to receive any help in dealing with their pain (that brought them to the dental office) for 6 months.

## Conclusion

The results of our study gave evidence that the SS provides some additional advantages over PS in the treatment of chronic TMD, especially in reducing symptoms of depression and improving pain-related disability. The efficacy of SS could be reflected in a significant decrease in oxidant/antioxidant ratio which was not present for PS. Furthermore, clinical success, in terms of reduction of depressive symptoms, which correlates with the reduction of oxidative stress markers in the SS group, indicates that oxidative stress might be related to psychological factors in TMD patients. If our results could be supported with randomized studies that are larger in the sample, the treatment for these common orofacial pain disorders could be understood better and, thus improved.

## Data Availability

The datasets generated and analyzed during the study are not publicly available, however, all data from this study is available from the corresponding author on reasonable request.

## References

[1] Chisnoiu AM, Pico AM, Popa S, Chisnoiu PD, Lascu L, Picos A, Chisnoiu R (2015). Factors involved in the etiology of temporomandibular disorders—a literature review. Clujul Med..

[2] Gauer RL, Semidey MJ (2015). Diagnosis and treatment of temporomandibular disorders. Am Fam Physician..

[3] Ohrbach R, Dworkin SF (1998). Five-year outcomes in TMD: relationship of changes in pain to changes in physical and psychological variables. Pain.

[4] Dersh J, Polatin PB, Gatchel RJ (2002). Chronic pain and psychopathology: research findings and theoretical considerations. Psychosom. Med..

[5] Tan G, Jensen MP, Thornby J, Sloan PA (2008). Negative emotions, pain, and functioning. Psychol Serv..

[6] Carlson CR, Okeson JP, Falace DA, Nitz AJ, Curran SL, Anderson D (1993). Comparison of psychologic and physiologic functioning between patients with masticatory muscle pain and matched controls. J Orofacial Pain..

[7] Fillingim RB (2011). Potential psychosocial risk factors for chronic TMD: descriptive data and empirically identified domains from the OPPERA case–control study. J. Pain..

[8] Macfarlane TV, Gray RJM, Kincey J, Worthington HV (2001). Factors associated with the temporomandibular disorder, pain dysfunction syndrome (PDS): Manchester case–control study. Oral Dis..

[9] Manfredini D, Winocur E, Ahlberg J, Guarda-Nardini L, Lobbezoo F (2010). Psychosocial impairment in temporomandibular disorders patients. RDC/TMD axis II findings from a multicenter study. J Dent..

[10] Quartana PJ, Buenaver LF, Edwards RR, Klick B, Haythornthwaite JA, Smith MT (2010). Pain catastrophizing and salivary cortisol responses to laboratory pain testing in temporomandibular disorder and healthy participants. J. Pain..

[11] Burris JL, Cyders MA, De LR, Smith GT, Carlson CR (2009). Posttraumatic stress disorder symptoms and chronic orofacial pain: an empirical examination of the mutual maintenance model. J. Orofac. Pain..

[12] Vasudeva S, Iyengar A, Seetaramaiah N (2014). Correlation of anxiety levels between temporomandibular disorder patients and normal subjects. J. Oral Dis..

[13] Fillingim RB (2013). Psychological factors associated with development of TMD: the OPPERA prospective cohort study. J Pain..

[14] Epker J, Gatchel RJ (2000). Coping profile differences in the biopsychosocial functioning of patients with temporomandibular disorder. Psychosom. Med..

[15] Garofalo JP, Gatchel RJ, Wesley AL, Ellis E (1998). Predicting chronicity in acute temporomandibular joint disorders using the research diagnostic criteria. J. Am. Dent. Assoc..

[16] Themessl-Huber M (2012). Weak evidence supports the use of psychosocial interventions for chronic orofacial pain. Evid. Based Dent..

[17] Costa YM, Porporatti AL, Stuginski-Barbosa J, Bonjardim LR, Conti PC (2015). Additional effect of occlusal splints on the improvement of psychological aspects in temporomandibular disorder subjects: a randomized controlled trial. Arch Oral Biol..

[18] Fricton J (2010). Systematic review and meta-analysis of randomized controlled trials evaluating intraoral orthopedic appliances for temporomandibular disorders. J. Orofac Pain..

[19] Basi DL (2012). Human temporomandibular joint and myofascial pain biochemical profiles: a case–control study. J. Oral Rehabil..

[20] Rodríguez Sotillo D, Velly AM, Hadley M, Fricton JR (2011). Evidence of oxidative stress in temporomandibular disorders: a pilot study. J. Oral Rehabil..

[21] Liu T, Zhong S, Liao X, Chen J, He T, Lai S, Jia Y (2015). A meta-analysis of oxidative stress markers in depression. PLoS ONE.

[22] Fedoce ADG, Ferreira F, Bota RG, Bonet-Costa V, Sun PY, Davies KJA (2018). The role of oxidative stress in anxiety disorder: cause or consequence?. Free Radic Res..

[23] Liu Z, Ren Z, Zhang J, Chuang CC, Kandaswamy E, Zhou T, Zuo L (2018). Role of ROS and nutritional antioxidants in human diseases. Front. Physiol..

[24] Gray RJ, Davies SJ, Quayle AA (1994). A clinical approach to temporomandibular disorders: a clinical approach to treatment. Br. Dent. J..

[25] Klasser GD, Greene CS (2009). Oral appliances in the management of temporomandibular disorders. Oral Surg. Oral Med. Oral Pathol. Oral Radiol. Endod..

[26] Kamodyová N, Tóthová L, Celec P (2013). Salivary markers of oxidative stress and antioxidant status: influence of external factors. Dis Markers..

[27] Alajbeg IZ (2013). Within-subject reliability and between-subject variability of oxidative stress markers in saliva of healthy subjects: A longitudinal pilot study. Dis. Markers.

[28] Schiffman E (2014). Diagnostic criteria for temporomandibular disorders (DC/TMD) for Clinical Research Applications: Recommendations of the International RDC/TMD Consortium Network and orofacial pain special interest Group. J.. Oral Facial Pain Headache..

[29] Von Korff M, Ormel J, Keefe FJ, Dworkin SF (1992). Grading the severity of chronic pain. Pain.

[30] Michelotti A, Iodice G, Vollaro S, Steenks MH, Farella M (2012). Evaluation of the short- term effectiveness of education versus an occlusal splint for the treatment of myofascial pain of the jaw muscles. J Am Dent Assoc..

[31] Löw B (2008). Validation and standardization of the generalized anxiety disorder screener (GAD-7) in the general population. Med Care..

[32] Spitzer RL, Kroenke K, Williams JB, Löwe B (2006). A brief measure for assessing generalized anxiety disorder: the GAD-7. Arch. Intern. Med..

[33] Kroenke K, Spitzer RL, Williams JB (2001). The PHQ-9: Validity of a brief depression severity measure. J. Gen. Intern Med..

[34] Baş B (2019). Effect of occlusal splint on interleukin 6, malondialdehyde and 8-hydroxydeoxyguanosine levels in synovial fluid of patients with temporomandibular disorders. Int. J. Oral Maxillofac. Surg..

[35] Dao TT, Lavigne GJ (1998). Oral splints: the crutches for temporomandibular disorders and bruxism?. Crit Rev Oral Biol Med..

[36] Bäck K, Hakeberg M, Wide U, Hange D, Dahlström L (2020). Orofacial pain and its relationship with oral health-related quality of life and psychological distress in middle-aged women. Acta Odontol Scand..

[37] Von Korff M, Simon G (1996). The relationship between pain and depression. Br J Psychiatry Suppl..

[38] Doron J, Trouillet R, Maneveau A, Ninot G, Neveu D (2015). Coping profiles, perceived stress and health-related behaviors: A cluster analysis approach. Health Promot Int..

[39] Vrbanović E, Lapić I, Rogić D, Alajbeg IZ (2019). Changes in salivary oxidative status, salivary cortisol, and clinical symptoms in female patients with temporomandibular disorders during occlusal splint therapy: a 3-month follow up. BMC Oral Health..

[40] Vrbanović E (2018). Salivary oxidant/antioxidant status in chronic temporomandibular disorders is dependent on source and intensity of pain—a pilot study. Front Physiol..

